# Supplementary material for: Ubiquitin Specific Protease USP48 Destabilizes NF-κB/p65 in Retinal Pigment Epithelium Cells
Source: Int J Mol Sci. 2022 Aug 26;23(17):9682. doi: 10.3390/ijms23179682 (PMC9456453; doi:10.3390/ijms23179682)
Supplement: Supplementary file 1 [file ijms-23-09682-s001.zip › ijms-1850542-supplementary.pdf]

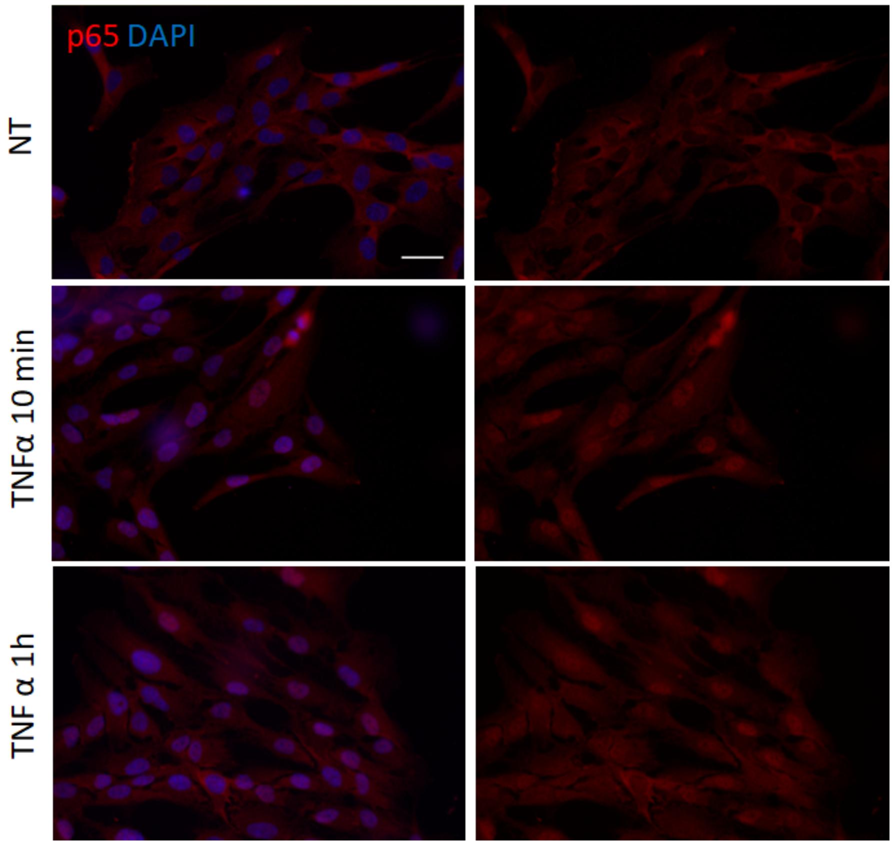

Figure S1. TNFα treatment induces efficient p65 nuclear localization in hTERT-RPE1 cells. p65 localization was assessed in hTERT-RPE1 cells at basal conditions and after treatment with TNFα at two different time points: 10 minutes and 1 hour to test the efficiency of nuclear translocation in stimulated cells. Scale bar: 40 μm.

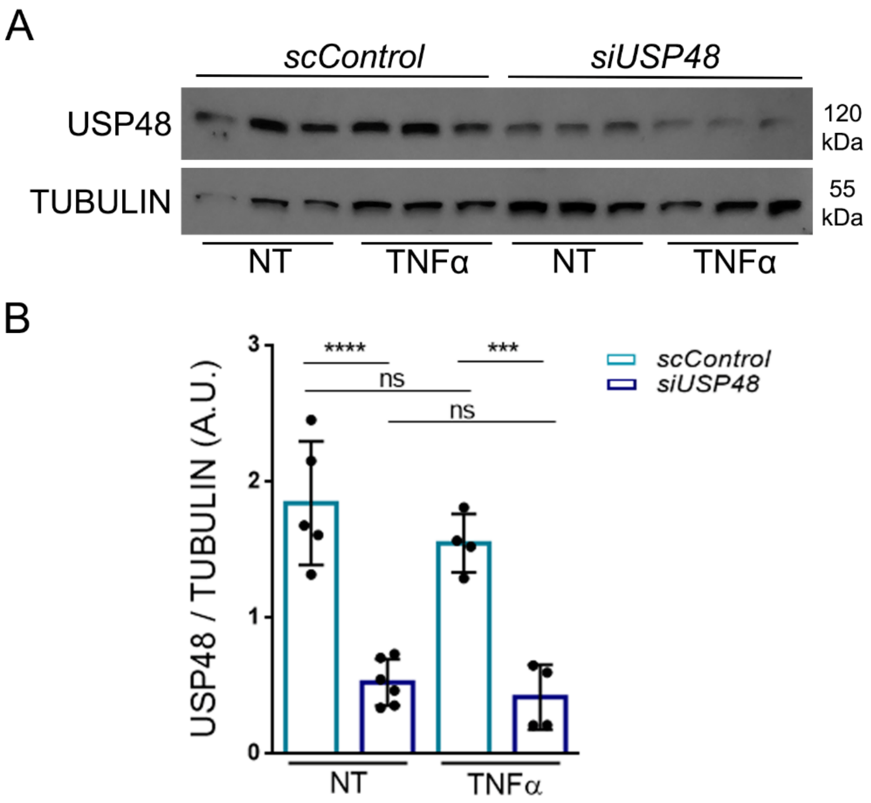

Figure S2. siRNA against *USP48* efficiently downregulates USP48 in both resting and TNF*α*-stimulated hTERT-RPE1 cells. (A) Cells were transfected with scrambled siRNA control (scControl) and siRNA against *USP48* (siUSP48), and 24 hours after transfection cells were treated with TNF*α* (4 hours). (B) Cell lysates were processed for western blot and densitometric quantification of USP48 was performed to confirm the efficiency of the knockdown, and that USP48 protein levels were not altered by TNFa stimulation. n = 4-6 samples from 3 independent experiments. Statistical analysis by One-Way ANOVA: *** p-value ≥ 0.001.

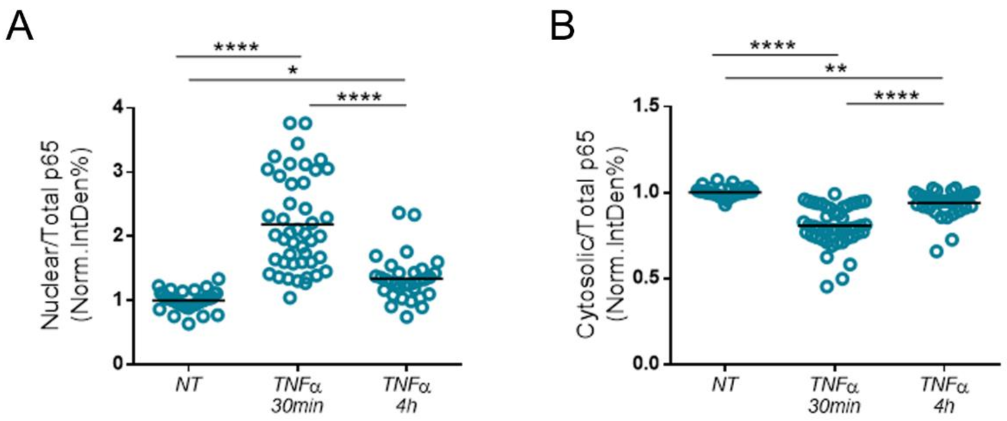

Figure S3. Nuclear/total p65 ratio increases after TNFα treatment. Nuclear/total p65 ratio increases after TNFα treatment, to the detriment of cytosolic/total p65 ratio in both the early response (30 minutes of treatment) and the late response (4 hours of treatment). Data are represented as the mean, n = 33-45 cells per condition from 3 independent experiments. Statistical analysis by One-Way ANOVA: * p-value $\geq$ 0.05. **** -value $\geq$ 0.0001.

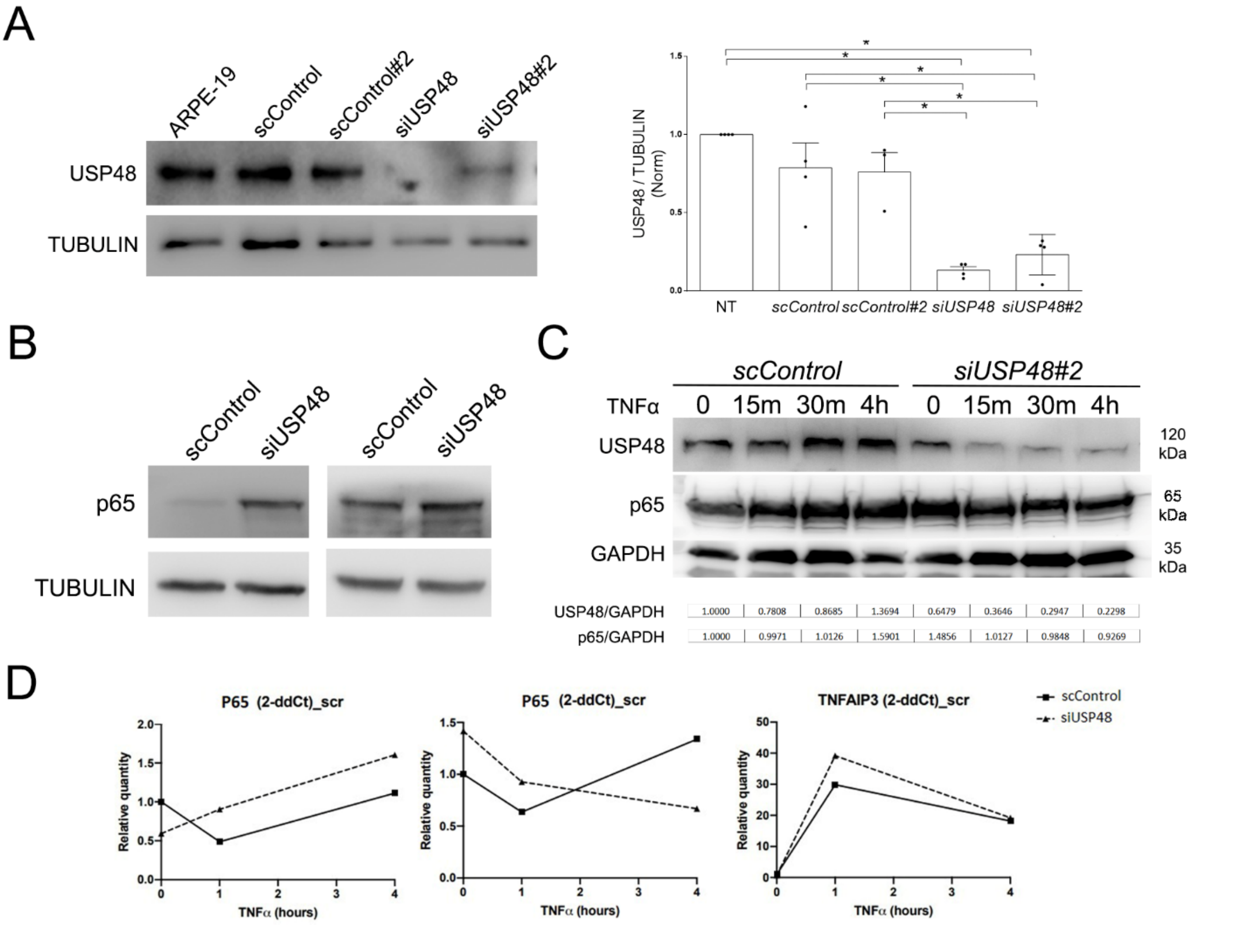

Figure S4. Assessment of USP48 knockdown upon treatment with two different specific siRNAs in two different retinal pigment epithelium cell lines, ARPE-19 and hTERT-RPE1, and effect on p65 levels. (A) ARPE-19 cells were transfected with 2 different scrambled siRNAs control (scControl or scControl#2), and 2 different siRNAs against USP48 (siUSP48 or siUSP48#2) to assay efficiency of *USP48* silencing. After 24 hours were processed for western blot and densitometric quantification of USP48 levels was performed to confirm the efficiency of the knockdown. Image represents one of 3-4 independent experiments. Normalized ratios USP48/TUBULIN ratios were calculated by densitometry. Statistical analysis by Mann-Withney: * p-value $\geq$ 0.05. (B) Silencing *USP48* in ARPE-19 cells (transfected with siUSP48) induces an increase in p65 protein levels. Two different replicates are presented. (C) Confirmation of both the efficiency of USP48 silencing and the increase effects on p65 protein levels were obtained in hTERT-RPE1 cells transfected with scrambled siRNA control (scControl) and siRNA against USP48 (siUSP48#2). After 24 hours, transfected cells were treated with TNFα. Cell lysates were processed for western blot at the indicated time points and densitometric quantification of USP48 was performed. Normalized ratios USP48/GAPDH and p65/GAPDH were calculated by densitometry and presented in the lower panel. (D) RT-PCR experiments showed that the levels of P65 transcript did not significantly change when USP48 was silenced. Results of 2 independent replicates are presented (left and middle graphs). NF-kB target gene TNFAIP3 was used as positive control (right graph).

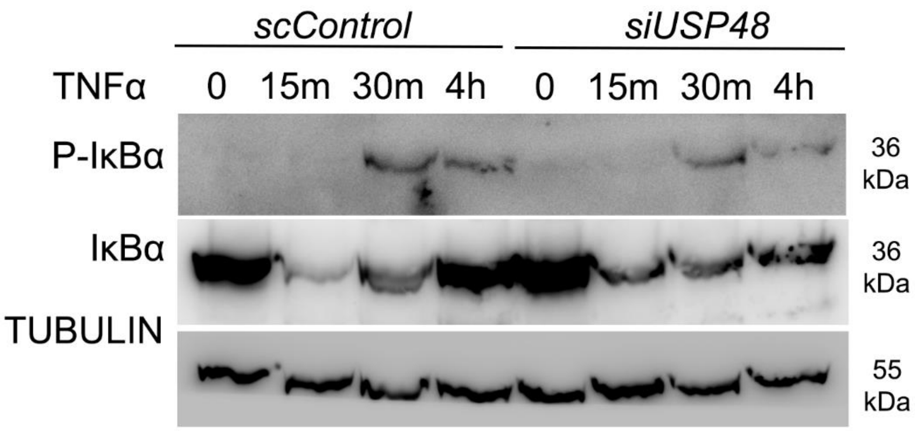

Figure S5. IκBα phosphorylation is not altered in USP48 downregulated cells. Following TNFα stimulation, IκBα is phosphorylated and degraded by the proteasome. hTERT-RPE1 cells were transfected with either scControl or siUSP48 and stimulated with TNFα. Cells were harvested at the indicated time point and lysed for western blot analysis. No major changes in the dynamic of IκBα phosphorylation and degradation were detected in USP48 downregulated cells when compared with control cells.

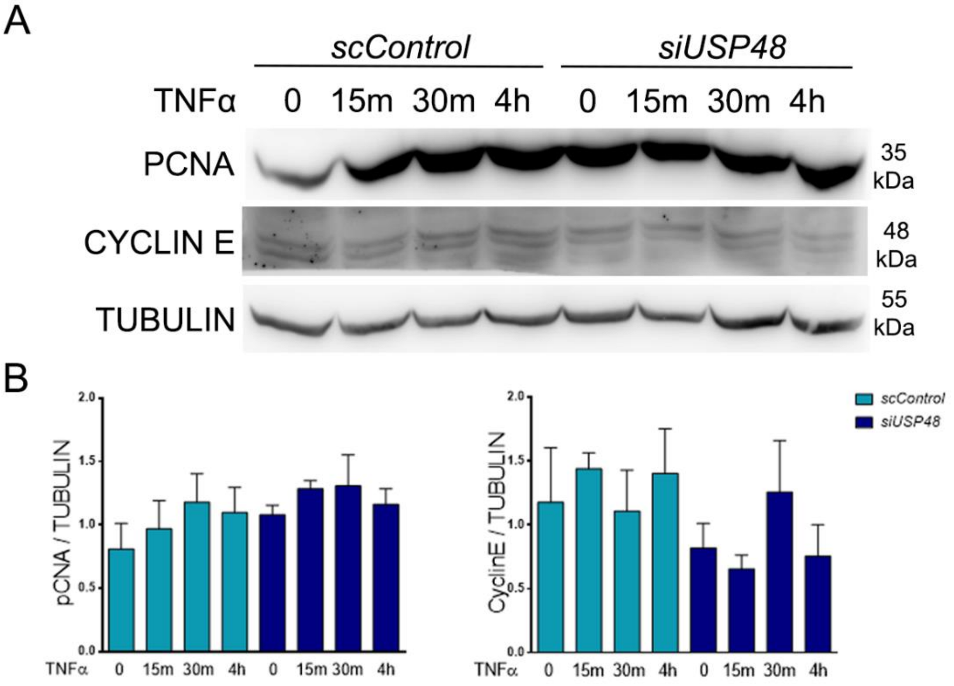

Figure S6. Knockdown of USP48 does not significantly alter protein levels of PCNA or Cyclin E (replication markers) with or without TNF*α* treatment. (A) Representative western blot on whole-cell lysates of hTERT-RPE1 cells transfected with scControl or siUSP48 and treated with TNF*α* at different time points. Tubulin was used as a loading control. Image represents one of 3-4 independent experiments. (B) Normalized ratios PCNA/TUBULIN and Cyclin E/TUBULIN ratios were calculated by densitometry. Although a slight increase in PCNA and a decrease in Cyclin E levels in non-treated (timepoint 0) USP48 knockdown cells seems apparent, these changes did not attain statistical significance.
